# Novel clinical, molecular and bioinformatics insights into the genetic background of autism

**DOI:** 10.1186/s40246-022-00415-x

**Published:** 2022-09-18

**Authors:** Ioanna Talli, Nikolas Dovrolis, Anastasis Oulas, Stavroula Stavrakaki, Kali Makedou, George M. Spyrou, Ioanna Maroulakou

**Affiliations:** 1 Department of Italian Language and Literature, School of Philosophy, Aristotle University of Thessaloniki, Thessaloniki, Greece; 2 Laboratory of Biology, Department of Medicine, Democritus University of Thrace, Alexandroupolis, Greece; 3 Bioinformatics Department, The Cyprus Institute of Neurology and Genetics, 6 International Airport Avenue, 2370 Nicosia, Cyprus, P.O. Box 23462, 1683 Nicosia, Cyprus; 4 The Cyprus School of Molecular Medicine, 6 International Airport Avenue, 2370 Nicosia, Cyprus, P.O. Box 23462, 1683 Nicosia, Cyprus; 5 Laboratory of Biochemistry, School of Medicine, AHEPA General Hospital, Aristotle University of Thessaloniki, Thessaloniki, Greece; 6 Laboratory of Genetics, Department of Molecular Biology and Genetics, Democritus University of Thrace, 68100 Alexandroupolis, Greece

**Keywords:** ASD, Genomics, Clinical phenotype, Genetic variation, Bioinformatics

## Abstract

**Background:**

Clinical classification of autistic patients based on current WHO criteria provides a valuable but simplified depiction of the true nature of the disorder. Our goal is to determine the biology of the disorder and the ASD-associated genes that lead to differences in the severity and variability of clinical features, which can enhance the ability to predict clinical outcomes.

**Method:**

Novel Whole Exome Sequencing data from children (*n* = 33) with ASD were collected along with extended cognitive and linguistic assessments. A machine learning methodology and a literature-based approach took into consideration known effects of genetic variation on the translated proteins, linking them with specific ASD clinical manifestations, namely non-verbal IQ, memory, attention and oral language deficits.

**Results:**

Linear regression polygenic risk score results included the classification of severe and mild ASD samples with a 81.81% prediction accuracy. The literature-based approach revealed 14 genes present in all sub-phenotypes (independent of severity) and others which seem to impair individual ones, highlighting genetic profiles specific to mild and severe ASD, which concern non-verbal IQ, memory, attention and oral language skills.

**Conclusions:**

These genes can potentially contribute toward a diagnostic gene-set for determining ASD severity. However, due to the limited number of patients in this study, our classification approach is mostly centered on the prediction and verification of these genes and does not hold a diagnostic nature per se. Substantial further experimentation is required to validate their role as diagnostic markers. The use of these genes as input for functional analysis highlights important biological processes and bridges the gap between genotype and phenotype in ASD.

## Background

According to the Diagnostic and Statistical Manual of Mental Disorders [1], Autism Spectrum Disorder (ASD) is associated with abnormalities in early developmental period in communication and social interaction and with restricted and repetitive patterns of behavior or interests. Cognitive skills such as intelligence, memory and attention, as well as language skills may also be affected in ASD. Forty-four percent of children identified with ASD has average and above average intellectual quotient (IQ > 85), 25% has below average IQ (71–85) and 31% is within the range of intellectual disability (IQ < 70) [2, 3]. Since ASD is a heterogeneous disorder, researchers usually adopt two main classifications: one based on the presence or absence of intellectual disability (ID) and one based on oral language skills (i.e., verbal or minimally verbal children).

The most common is the one that classifies the following two main subgroups: those that ASD coexists with intellectual disability and those that have average or above average intellectual functioning, whose characteristics vary in terms of linguistic, cognitive and social skills from those with intellectual disability [4]. Another classification for children within the autistic spectrum is verbal and non-verbal or "minimally verbal" children, i.e., children who have very limited use of spoken language for communication purposes. The reception of language might also be affected, and the autistic symptoms are usually severe in terms of behavior [5–8]. It has been commonly believed that non-verbal cognitive abilities predict expressive and receptive language [2, 3]. However, Hanson et al. [9] have shown that there are minimally verbal children with autism who do not have low non-verbal IQ, others with low both expressive and receptive language skills and others that have low expressive but good receptive language skills. Consequently, categorization of subgroups in ASD is problematic.

Additionally, the association of genetic loci with specific behavioral characteristics in ASD contributes significantly to the understanding of the influence of genetic factors on clinical phenotype. This connection arises from studies that in their methodology include, in addition to genetic analysis, behavioral assessment, such as language and cognitive assessment. Recently, various researchers have suggested that genetics provide a lot of information on clinical phenotypes of ASD rather than vice versa [6]. Several chromosomal copy number variants (CNVs) and single-nucleotide variants (SNVs) (such as deletions and duplications at chromosomal regions 1q21, 7q11.23, 15q11–13, 16p11.2, and 22q11.2) have been identified as genetic risk factors for ASD [7, 8] and have shown to have predictive value for clinical phenotype of ASD [5]. For example, 15q11.2 duplications are linked to ASD and Schizophrenia [10] in addition to their connection to high rates of epilepsy [11]. Other deletions, including 16p11.2, have been linked to cognitive deficits such as intellectual disability [9, 12] as well as developmental coordination disorder, phonological processing disorder, expressive and receptive language disorders [13]. Prognostication of the clinical profiles of individuals with ASD based on specific genes that could serve as reliable biomarkers is important for early diagnosis and eventually for early and effective treatment. These findings would not be possible without contemporary sequencing and bioinformatics methods.

The technological advancements of next-generation sequencing (NGS), including whole genome sequencing (WGS) and whole exome sequencing (WES), have enabled researchers to perform detailed gene variation analyses like genome-wide association studies (GWAS) en masse. This newfound accessibility to these technologies enables not only experimental high-throughput protocols to be undertaken but also provides clinicians with powerful tools for assessing disease pathogenesis, progression and outcome. It has also enabled clinicians to provide more gene guided counseling into matters like therapy (through pharmacogenomics), and pre/peri-natal consulting. However, there are a variety of factors that need to be taken into account especially due to the complex nature of various diseases and the idiosyncrasies of individual patients regarding their genetic background. These notions bring forward precision medicine.

In precision medicine, genetic variation screening provides an important tool for detecting high-risk individuals of specific genetic disorders. Odds ratio analysis employed in traditional GWAS helps ascertain disease-variant associations by the occurrence frequency of these high-risk variants in non-control groups. These variants can act both protectively and as instigators of disease. To make this determination, researchers can employ polygenic risk score predictions by training risk models on pools of variants highlighted in specific case–control studies [14, 15]. Alternatively, variant annotation using in-silico approaches like GEMINI [16] provide information for each variant found in a study’s samples through several genomic databases (ENCODE [17], UCSC [18], OMIM [19], dbSNP [20], KEGG [21], and HPRD [22]) and informs on frequency (like ExAC [23] and 1000GP [24]) and proteinic impact of changes in amino acid coding due to these variants (ClinVar [25], COSMIC [26], CADD [27], Polyphen [28] and SIFT [29]).

Current WHO criteria for classification and grading of ASD provide a valuable but simplified depiction of the true nature of the disorder. Moreover, it is often difficult to predict clinical outcome using the current grading scheme. The aim of this study is to elucidate, through clinical assessment and bioinformatics, the differences in the genetic background of different phenotypical manifestations of ASD. More specifically, it aims at investigating whether there are specific genes that can account for differences in the clinical profiles of children with ASD at the linguistic and cognitive level by reporting on the analyses of a new autistic patient WES dataset (*n* = 33). We first extracted the sequenced genotypes (WES) of blood samples of school-aged (6–12-year-old) children with ASD. We then conducted clinical assessment by administrating standardized tests of non-verbal IQ, memory, attention and oral language skills and separated them into mild and severe phenotypes in each of these cognitive and linguistic categories, based on these assessments. The next step was to identify common high-risk variants in the sample dataset previously found in the literature by searching through several genomic databases but perhaps also to identify de novo variants, not previously reported in the literature. Finally, we used a linear regression polygenic risk score machine learning algorithm to obtain biologically significant genes with the potential to aid in the grading of autistic samples based on their sequenced genotypes, derive specific molecular signatures from severe and non-severe subtypes of autistic samples and assess whether these molecular signatures outline functional subclasses. At this stage we should stress that given the limited number of patients in the dataset used in our study, results require additional validation using further experimentation. With this in mind, we report eighty-four identified variants which could be assigned to specific functional categories related to ASD and intellectual disability, as well as other disorders. Classification of our samples using these variants was in agreement with the clinical classification for our dataset with 81.81% prediction accuracy. The six samples that showed a differential molecular diagnosis were further assessed using clinical information in order to substantiate the classification provided by our risk model.

## Methods

### Participants

Thirty-three children with ASD that attended both mainstream and special education schools were recruited from private speech therapy centers. Only those children whose parents gave written permission to participate in the research were included in the study. All children were diagnosed with ASD by public hospitals and public medical-pedagogical centers according to the ICD-10 (https://apps.who.int/iris/handle/10665/37958) and DSM-V [1] official criteria. Children were initially divided into two groups based on their non-verbal IQ. The first included 18 children (average age: 9.5 years) with typical non-verbal IQ (> 80 in Raven Progressive Matrices) (ASD_MH group) and the second included 15 children (average age: 8.5 years) with low non-verbal IQ (< 60 in RPM) (ASD_L group). We then divided them based on whether they were verbal (acquired spoken language) or minimally verbal (absence of spoken language) children with ASD. The criterion was their performance (score 0) in two language tasks that required spoken language (see below expressive vocabulary and narration tasks). There were 19 verbal and 14 minimally verbal children. Moreover, we divided them in two groups (severe and mild) based on their attention and memory skills. Regarding the attention skills, the criterion for a child to fall under the severe phenotype was performance under the 10th percentile in both auditory and visual attention tasks and equal or over the 10th percentile for the mild phenotype. There were 9 children in the mild and 24 in the severe phenotype concerning attention skills. Regarding the memory skills, the criterion for a child to fall under the severe phenotype was performance under the 10th percentile in both auditory and visual memory tasks. There were 18 children in the mild and 15 in the severe phenotype.

Participants were assessed at their school individually in one or two sessions of a total duration of 45 min. Moreover, blood samples were obtained by experienced microbiologists in microbiology laboratories and were then sent to a genetic lab for Whole Exome Sequencing analysis.

### Clinical phenotype assessment

In this study, children were assessed with cognitive as well language tasks. Standardized tests for Greek were employed. More specifically, our assessment materials included:

#### Cognitive measures

Non-verbal IQ. Non-verbal IQ was assessed with the Greek version of Raven Standard Progressive Matrices [30, 31]. Both standard scores and percentiles were taken into consideration.

Auditory and visual attention. Auditory and visual attention was assessed using three subtests of the Test for the Assessment of Attention and Concentration [32] (i) Sustained auditory attention, (ii) Sustained visual attention and (iii) Range of visual attention. The Total Attention Score of all three auditory and visual attention subtests was also calculated.

Verbal short-term memory, visual and auditory memory. There were totally seven measures: VSTM Sentence recall [32], VSTM word recall [33], Immediate visual memory, Delayed visual memory, Visual information recall, Information retention factor (Story Recall subtest of the Memory Test; see Narration below) and Recognition.

#### Language measures

Expressive vocabulary. It was assessed using the Greek version of Crichton Vocabulary Scales [31]. It contains 80 word definitions, presented orally, and arranged in order of increasing difficulty (interruption criterion: four consecutive errors). Only one child from the ASD_L group was able to name a few definitions, so for all the rest of the children in the ASD_L group, Picture Naming and Comprehension Subscale was administered.

Picture Comprehension. It was administered only to the ASD_L group, because all but one were minimally verbal. Receptive vocabulary was assessed using Picture Comprehension Subscale (Detection of Speech and Language Disorders Test Preschool, [DSLD Test] [34]), in which the child was asked to point to the picture (among 4) that corresponded to the word presented orally by the examiner.

Narration. Narration was assessed by using the Story Recall subtest of the Memory Test [33]. The child would listen to two short stories and repeat them back right after the examiner and after a short break (scoring: total number of elements and total number of sections s/he remembered correctly).

### Sequencing, mapping, alignment and variant calling

Exome enrichment library was prepared with the Agilent SureSelectXT Human All Exon V6 kit as per the manufacturer’s instructions. Read files (Fastq) were generated from the sequencing platform (Illumina Hiseq). The samples were sequenced in paired end, 2 × 100 bp mode and deep coverage was obtained with approx. 6–7 Gb per sample (approx. 100 × av. coverage). Quality assessment and trimming was performed using the FastQC version: 0.11.7 and FASTX version: 0.0.14 toolkits, respectively. The Burrows-Wheeler Aligner (BWA) [35], version: 0.7.15 was used to map the raw reads to the human genome (build hg19/b37). Duplicate reads, which are likely to be the results of PCR bias, were marked using Picard (http://broadinstitute.github.io/picard/) version: 2.6.0. Samtools [36], version: 0.1.19, was used for additional BAM/SAM file manipulations. The Genome Analysis Tool Kit (GATK) [37], version 3.6.0, Haplotype Caller method was used for single-nucleotide polymorphism (SNP) and insertion/deletion (indel) variant calling.

#### Variant annotation

Variants were annotated with gene functional data from Ensembl version 90 using the Variant Effect Predictor (VEP) tool, version 90.6. Known variants were labeled using the dbSNP (Release 147) allowing for rapid identification of novel variants. Additional exploration of the results was performed using GEMINI, version 0.20.0, which provides a framework for analyzing, filtering and exploring genomic variation.

#### Odds ratio analysis

PLINK [38] was used to perform odds ratio analysis for obtaining variants with high disease association. PLINK allows for the detection of variants more frequently associated with severe than non-severe cases, which are labeled as high-risk or pro-severe variants. In contrast variants being more frequently associated in non-severe than severe cases are labeled as protective or pro-non-severe variants.

#### Risk score prediction using linear logistic regression analysis and risk model construction

Linear logistic regression fitting was performed using the PredictABEL [39] package (available in R). Specifically risk models were constructed using the fitLogRegModel function, and the predRisk function was used to assess their performance and predict risks. Additional functions available by the package were used for the various measures to assess model performance. Commonly in genetic risk prediction studies this includes: (i) plotting receiver operating characteristic (ROC) curves and calculating area under the curve (AUC) values using the plotRoc function and (ii) the reclassification table construction, net reclassification improvement (NRI) and integrated discrimination improvement (IDI) calculations using the reclassification function. The NRI and IDI are important comparative measures that provide an assessment of how well a new model reclassifies the data [40]. Graphical representation of results were attained using the plotRiskDistribution function for plotting risk distributions, the plotDiscriminationBox function for plotting discrimination box plots and the plotPredictivenessCurve function for plotting predictiveness curves. Better model performance was achieved by substituting the glm (generalized linear model) function utilized by PredictABEL with the bayesglm function available from the arm R package [41].

### Machine learning data analysis

**Fig. 1:**
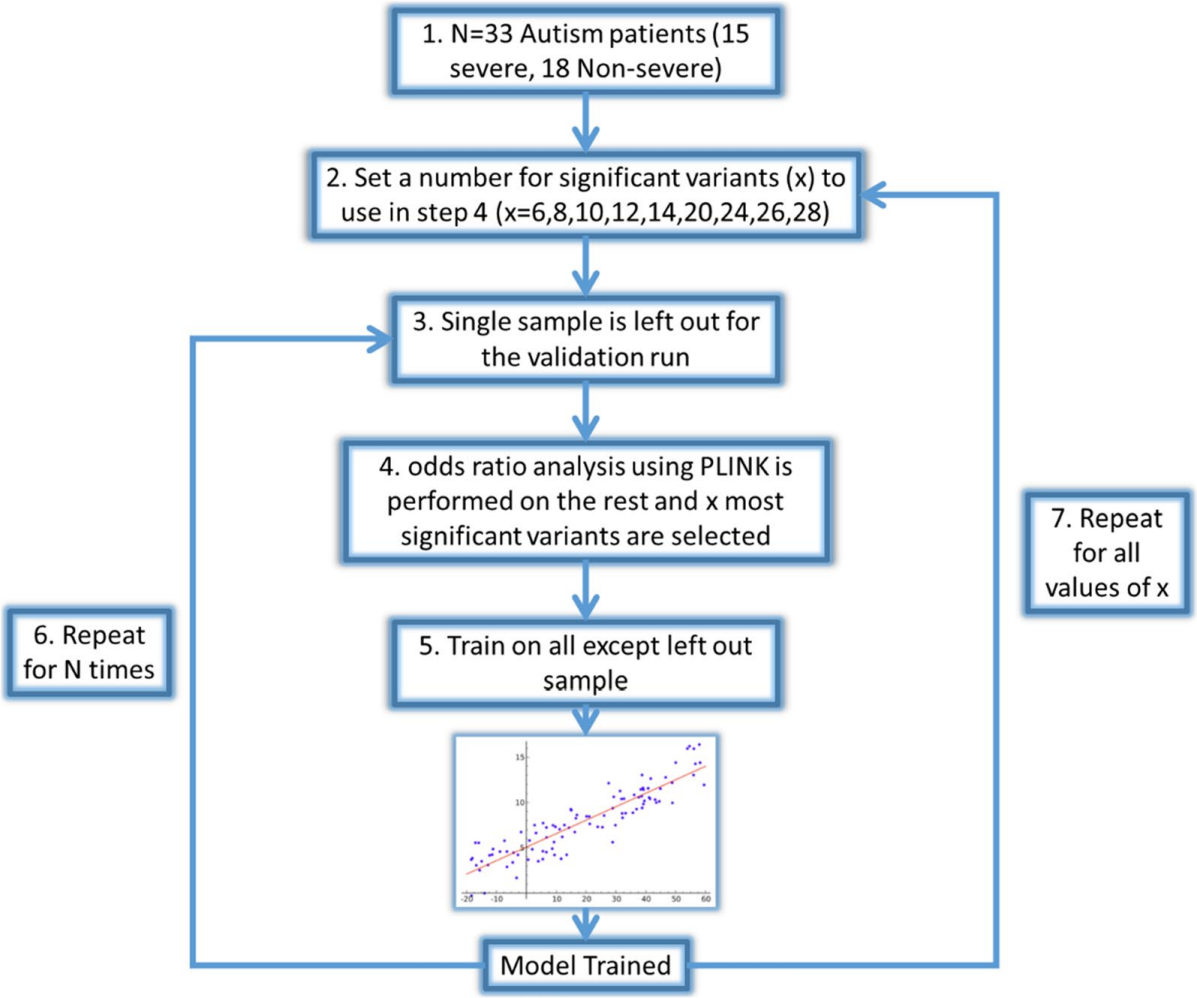
Flowchart of the classification process suing a linear regression classifier and PLINK as the feature (variants) selection tools. Step 1—Describes the dataset with 2 classes of patients with Severe and Non-severe autism. Step 2—Denotes the selection of a set of variants to be used during the leave-one-out classification (LOOCV) process. Step 3—The LOOCV is initiated by extracting one sample from the dataset. Step 4—PLINK is used to perform odds ratio analysis on the remaining samples (this avoids overfitting). X number of variants top significant variants are used for the next. Step 5—A linear regression model is trained using the data and features for the specific iteration. Step 6—the LOOCV process (Steps 3–5) is repeated for every sample (N = 33) and statistics recorder. Step 7—Steps 2–5 are repeated for every value of X

The samples obtained were separated into 2 classes based on their non-verbal IQ representing severe autism (n = 15) and non-severe autism (n = 18) and concurrently used as input for training the linear regression model classifier described above. Assessment of classifier performance was achieved using a leave-one-out cross-validation (LOOCV) procedure. During each round of cross-validation, each sample was removed recursively and feature selection was performed on the remaining samples in the dataset, the model was then trained and utilized to classify the left-out sample. For the feature selection (variants), PLINK was used. To find the optimal set of variants, the leave-one-out cross-validation method was performed by testing initially the top six variants and sequentially increasing the number of variants for each run until classification accuracy reached a saturation point with no further improvement. LOOCV performance was assessed using prediction accuracy, sensitivity, specificity, and Matthew’s correlation coefficient (MCC). MCC is defined as a balanced measurement of the classification quality which takes into account true and false positives and negatives. MCC returns values within the range of [− 1, 1]. The flowchart of the classification procedure is shown in Fig. 1.

### Literature-based genomics approach

To complement our de novo classification approach, which predicts if a sample fits into the two main autism measurements under investigation (mild and severe), we utilized a novel pipeline for the identification of genes which characterize the IQ, verbal, memory and attention measurements based on current scientific knowledge. This pipeline consists of several distinct steps:

Creating a subgroup of the variants in each of our samples which exhibit homozygosity to the alternate allele of our reference.

From the previous subgroup discarding any variant which isn’t flagged simultaneously in the SIFT [29] (database as “deleterious” or “deleterious_low_confidence” and in the Polyphen [28] database as “possibly_damaging” or “probably_damaging”. The remaining genes were deemed “important” (IGs).

Keeping only the above variants for each sample, divide the samples into sub-phenotype groups based on our original phenotypical assessment measurements.

Running all samples of a sub-phenotypical group through R’s SuperExactTest [42] package to identify genes common among at least n-2 samples (where n is the total number of samples in each category).

**Fig. 2:**
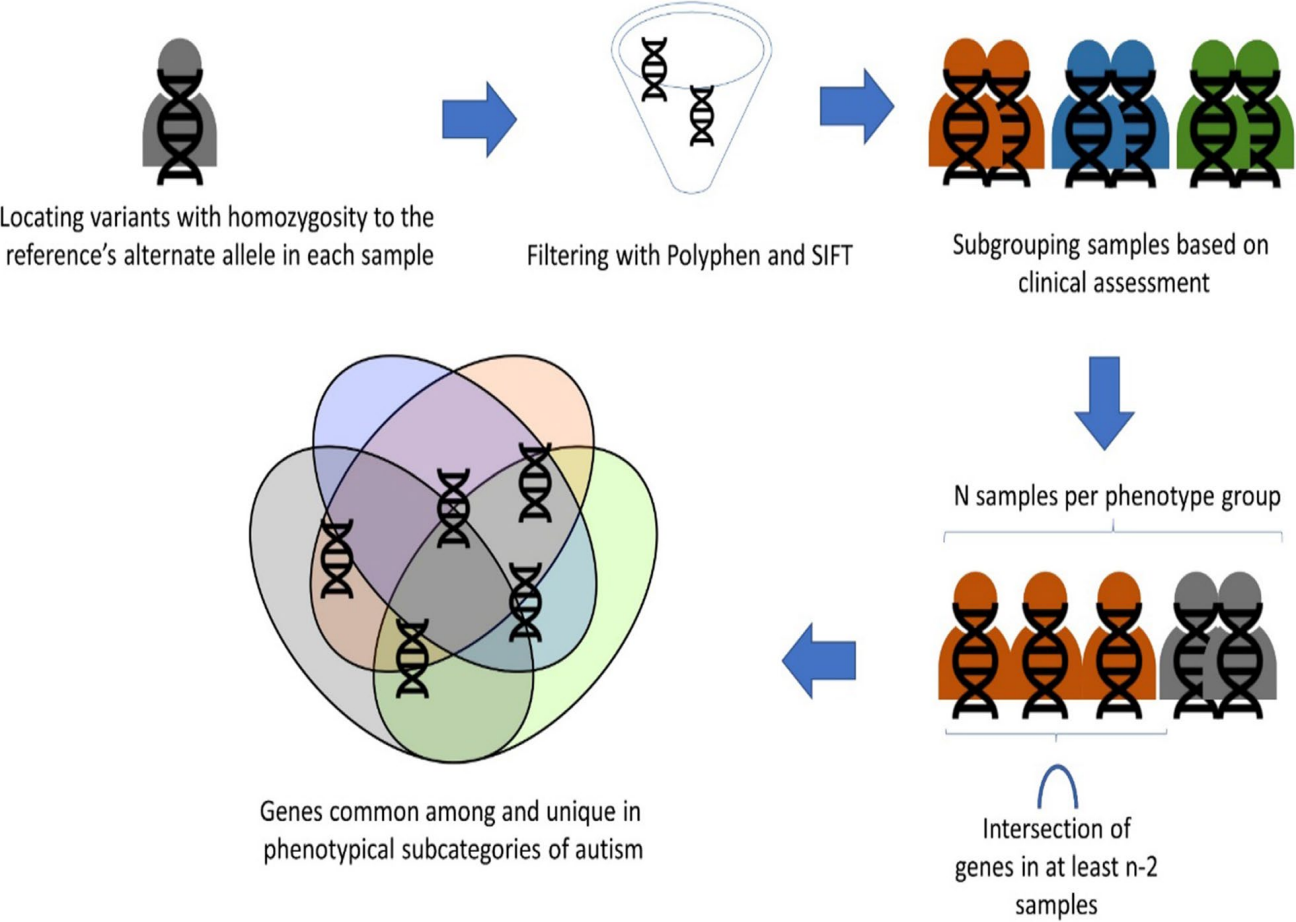
Pipeline for the identification of genes which characterize the IQ, verbal, memory, and attention measurements based on current scientific knowledge provided by the databases SIFT and Polyphen

Finally, comparing the genes in each sample grouping via VENNY [43] to identify genes which characterize a group but also genes which are common among them and describe a more generic autism genetic signature. This approach is visualized in Fig. 2.

### Validation gene dataset

To validate our results versus genes that are already known in literature in connection to autism, we created a gene dataset by extracting all autism-related genes from 5 databases (AutismKB [44], SFARI [45], HuVarBase [46], DisGeNET [47] and OpenTargets [48]), on which, using Venn Diagrams, we superimposed the results of our 2 approaches. This also allowed to identify genes that were found de novo as being implicated in autism by our study.

### Functional analysis

To better understand the pathways and mechanisms involved in Severe-Mild autism classification and sub-phenotyping, we performed a variety of functional analyses on our gene data from both approaches. REACTOME [49] was used for the first pass identifying genes involved in various pathways. The genes not found in REACTOME were manually researched in literature and other sources like GeneCards [50], STRING [51], Uniprot [52], Mammalian phenotype Ontology [53] and Gene Ontology [54] for their functional associations.

## Results

Results of the performance of the two groups according to our clinical measures on the experimental tasks are presented in Table 3 as well as between group comparisons. For the different tasks (all except picture comprehension), non-parametric tests (Mann–Whitney test) were conducted to compare performances of ASD_MH and ASD_L groups.

### Phenotypic results

#### Cognitive measures

As expected by the inclusion criteria of the groups, in non-verbal IQ ASD_L group had worse performance (Mann–Whitney U = 0, *p* < 0.001). The same holds for attention total score, auditory attention, visual attention and visual range attention (Mann–Whitney U = 13, 24, 7 and 20.50, respectively, *p* < 0.001). In VSTM sentence and word recall the difference between the two groups was also significant (Mann–Whitney U = 11.50 and 4 respectively, *p* < 0.001), as well as in immediate and delayed visual memory, visual information recall, information retention factor and recognition (Mann–Whitney U = 13.50, 24, 32, 32 and 10 respectively, *p* < 0.001).

#### Language measures

In expressive vocabulary, the difference between the two clinical groups was significant as expected (Mann–Whitney *U* = 0, *p* < 0.001). In narration, the difference in groups' performance was also significant in both total elements and total sections (Mann–Whitney *U* = 25.50 and 32, respectively, *p* < 0.001).

In sum, in all measures, both cognitive and language, the ASD_MH group outperformed the ASD_L group.

### Machine learning data analysis

**Fig. 3:**
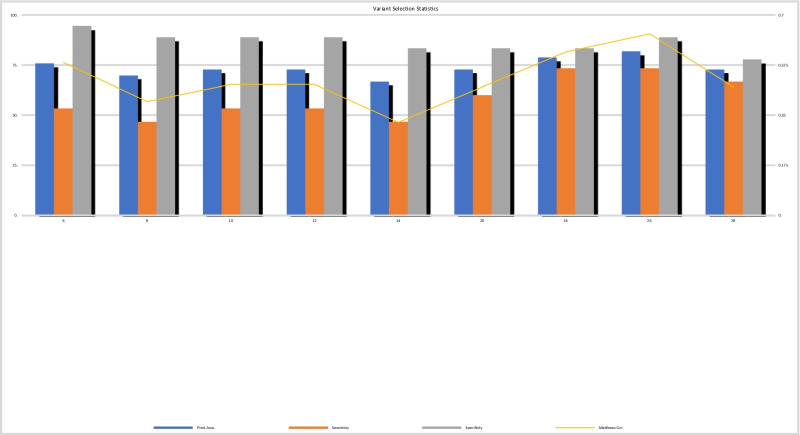
Results of the Classification process described above showing statistics for each LOOCV run across different values of top significant variants selected for validation. Statistics are recorded in the form LOOCV prediction accuracy (blue bars) of sensitivity (orange bars) specificity (gray bars) and finally Matthews correlation is shown (yellow line)

**Fig. 4:**
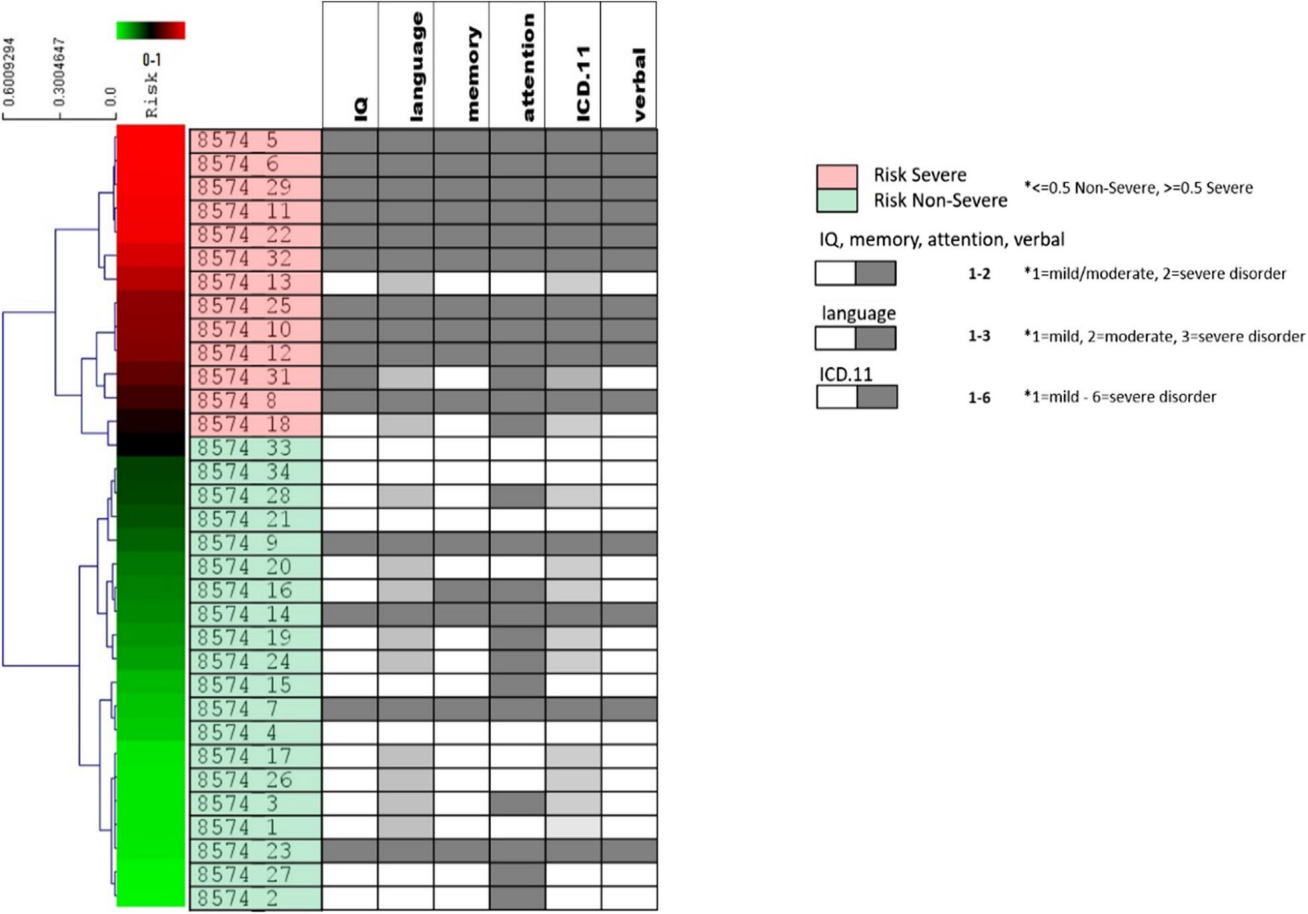
Visualization of risk model results for 33 ASD patients (18 non-severe and 15 severe) using the 26 variants selected during LOOCV. The dendrogram was obtained by performing hierarchical clustering (using Euclidean distance and average linkage algorithm) of model prediction outputs. The clustering represents the molecular subtypes obtained by the trained model for all ASD patients. The two molecular subtypes as predicated by the risk models are color-coded as pink for the most severe cases (high-risk individuals), light green for least severe cases (low-risk individuals). Moreover, the continuous spectrum of risk prediction scores is shown in the red-green gradient traversing the dendrogram. Patients are further sorted by severity in descending order. Clinical experimental data is also viewed in parallel to the results obtained from the machine learning algorithm and are shown as columns with dark and light gray boxes. The boxes denote the different level of severity for the six different clinical data available for this study. The molecular classification of samples 8574_9, 8574_14, 8574_7 and 8574_23 appears to differ from the clinical classification. These samples cluster separately from the rest of the samples with similar severe clinical phenotypes. Similarly, based on theory molecular classification, samples 8574_13 and 8574_18 also appear to cluster away from samples of similar non-severe clinical classification

Machine learning was performed by utilizing the severe autism (*n* = 15) and non-severe autism (*n* = 18) samples to train the linear regression model classifier described in the Material and Methods. As detailed in Fig. 1, a leave-one-out cross-validation (LOOCV) procedure was used for to assess the classifier performance. Feature, or variant selection was coupled to the LOOCV procedure to ensure an optimum set of best classifier variants is obtained. The optimum set was determined to be the top 26 variants for every LOOCV iteration and therefore these variants were selected for downstream functional analysis. To assess the performance of each feature selection run, the accuracy, specificity, sensitivity and Matthew’s correlation coefficient (MCC) were calculated. Results are summarized in Fig. 3. Comparison of the optimum results attained by the molecular subtype classification defined by our risk model, with prior clinical grading, showed that they were in agreement with 81.81% (27/33 samples) prediction accuracy. Sensitivity, specificity and MCC achieved values of 73.33%, 88.89% and 0.634, respectively. Plotting receiver operating characteristic (ROC) curves resulted in area under the curve (AUC) with value 0.83. Visualization of risk model outputs for all samples using clustering algorithms (including annotation with clinical metadata) is shown in Fig. 4. The top 26 variants obtained from every LOOCV iteration were pooled together to obtain a total of 84 unique variants. Table 4 shows a list of these variants as well as their genes and full annotation including the frequency of occurrence according to the 1000 genome project (aaf_1kg_all). Full annotation for these variants including levels of heterozygosity and homozygosity and annotation in clinical database such as ClinVar. Notable molecular significant variants from the list are known to be implicated in the genetic predisposition of certain diseases and disorders including: certain cardiomayopathies (rs12063382), hypertension (rs1061157), afibrinogenemia (rs2070018), ciliary dyskinesia (rs3752042), congenital cataract (rs4682801), prostate cancer (rs1328285, rs9890913), infantile epilepsy and Parkinson’s (rs56260729), mental disability and schinzel-giedion syndrome (rs12922670, rs11082414), cerebellar hypoplasia (rs77247739), kabuki syndrome (rs5952285, rs5952682), autism (rs7049300) and even response to drug administration such as ezetimibe (rs10264715).

In addition to the variant biological insights, the same thought process can be applied on the genes themselves. In total this method highlighted 60 genes, 12 related to mild and 48 to severe autism. Out of those 3 related to mild autism and 12 related to severe autism are already known and can be found in the validation dataset created from 5 databases which is described in our methodology. These results can be found in Table 1.

**Table 1:** Validation of the IGs highlighted by our machine learning approach with the help of the 5 autism-related databases (AutismKB, SFARI, HuVarBase, DisGeNET and OpenTargets)

Severity	Novel	Known (5 database validation set)
Mild	*n* = 9	*n* = 3
	*FYCO1, MROH2B, ZNF131, KIAA1456, CCDC171, ZFC3H1, CCDC38, COG3, TJP1*	*COL11A1, FGA, NCOA6*
Severe	*n* = 36	*n* = 12
	*AGRN, C1orf222, LRRC71, ACTN2, AGXT, AC104809.3, PRSS50, TNK2, NMUR2, MRPL22, C6orf229, HIST1H1A, IQCE, NPC1L1, OR2A12, OR2A2, ANKRD18B, C9orf84, NDOR1, OR6M1, TTC6, ANXA2, SLX4, ACSM1, TSEN54, ENGASE, CCDC40, MED16, ZNF431, USF2, CCDC114, ZNF813, BPIFB6, BPIFB4, ZBP1, XG*	*ARHGEF11, BMPR2, NGEF, SSPO, NAT2, MMP16, CDH15, ASIC2, SETBP1, NLGN4X, NR0B1, KDM6A*

### Literature-based approach—IGs

Using the approach previously described in our methodology, 1005 unique genes were evaluated as being homozygous to the reference alternate allele and marked in the SIFT and Polyphen databases as IGs in all our samples. Before focusing on sub-phenotypes we just pooled the IGs from children with mild and severe autism, respectively, together and validated this IGs dataset versus the 5 databases (See Methodology). In total 96 IGs of mild autism and 98 IGs of severe autism were found in the validation dataset. Out of those 70 were common between them.

**Fig. 5:**
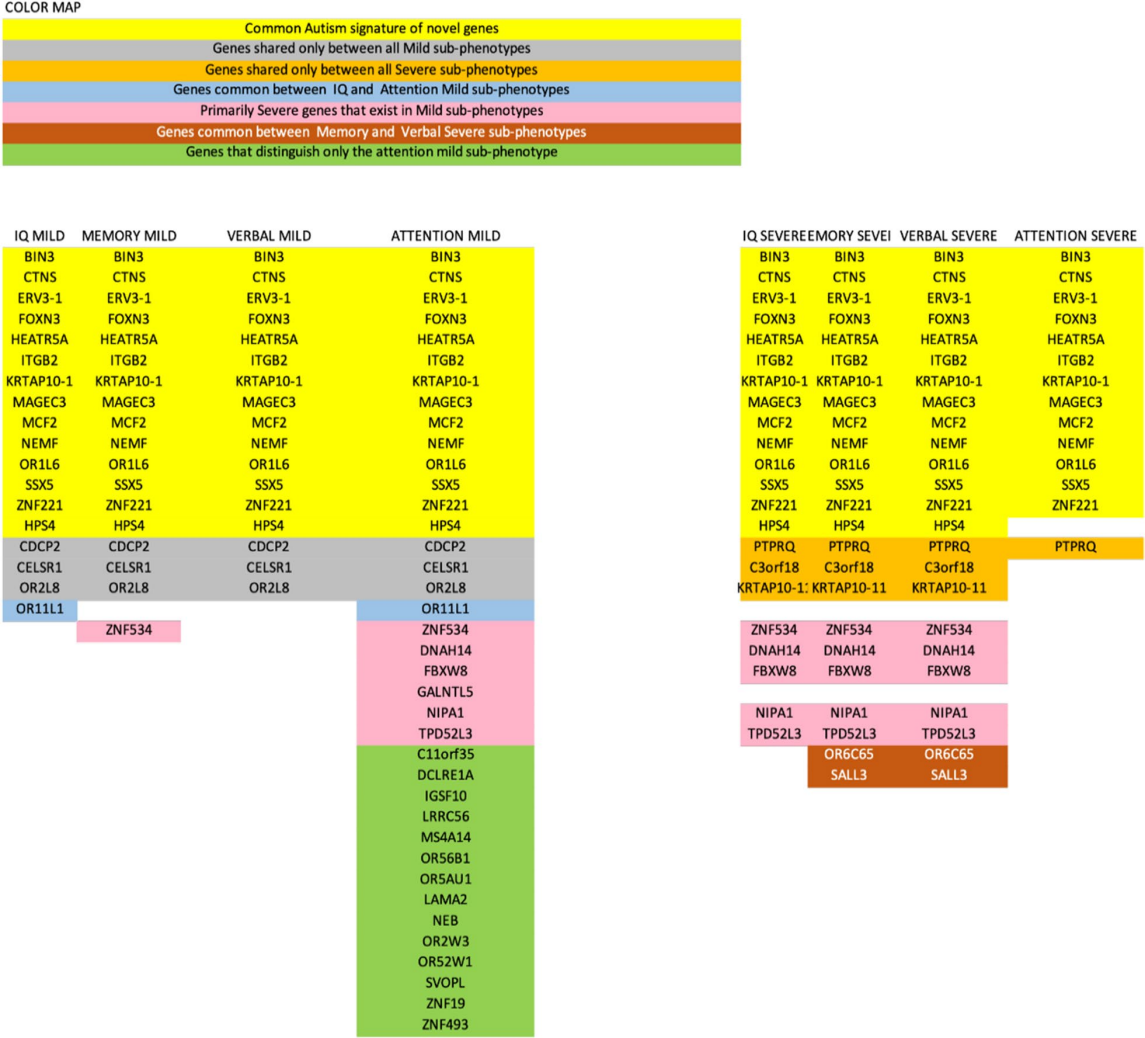
Common and distinct selected Genes harboring variants (candidate mutations) with possible contribution to ASD risk, based on severity across distinct ASD subgroups (phenotypes)

Investigation of the sub-phenotypical sample clusters of IQ, Memory, Attention, and Verbal, based on the clinical observations, led to identifying 14 IGs which were present in all sub-phenotypes regardless of severity (Fig. 5 in yellow background).

In samples from children with mild autism, the “IQ mild” sub-phenotype had 18 IGs common in all samples of the group, the “Memory mild” 18 IGs, the “Verbal mild” 17 IGs and the “Attention mild” 38 IGs. Also 3 IGs (*CDCP2*, *CELSR1*, *OR2L8*) were common between all mild sub-phenotypes. The “IQ mild”, “Memory mild” and “Verbal mild” sub-phenotypes were almost identical, with the exception of *OR11L1* for “IQ mild” and *ZNF534* for “Memory mild” being highlighted as IGs. *OR11L1* was missing as IG for “Memory mild” and “Verbal mild” but was highlighted in “Attention mild”. There is also a group of 7 genes (*C11orf35*, *DCLRE1A*, *IGSF10*, *LRRC56*, *MS4A14*, *OR56B1*, *OR5AU1*) which were highlighted as IGs only in “Attention mild”.

Samples from children with severe autism when studied per sub-phenotype highlighted 22 IGs for the “IQ severe group”, 24 IGs for “Memory severe”, 24 IGs for “Verbal severe” and 14 IGs for “Attention severe”. The IG *PTPRQ* was found in all our severe autism samples. The “Memory severe’ and “Verbal severe” sub-phenotype IGs were identical. In addition, the IGs *OR6C65* and *SALL3* were only found in these 2 sub-phenotypes. IGs *KRTAP10-11*, *ZNF534*, *DNAH14*, *FBXW8*, *NIPA1*, *TPD52L3* were common in all severe sub-phenotypes with the exception of “Attention severe”, whereas *C3orf18* was found as IG only in “IQ severe”, “Memory severe” and “Verbal severe”. Finally, the “Attention severe” group was the only one lacking the *HPS4* severity-independent IG.

Another round of validation versus the 5 database dataset was performed for these sub-phenotype IGs (Table 2). In total 10 IGs exist in both our data and the validation dataset. Their breakdown per sub-phenotype is: *NEMF* was highlighted for all sub-phenotypes regardless of severity. *NIPA1* was highlighted in all severe sub-phenotypes except for “Attention severe” in which it was found in 87% of samples, and all samples of “Attention mild”. *CELSR1* was validated for all mild sub-phenotypes. *MS4A14* was validated only in “Attention mild”. Finally, “Attention mild” was the only sub-phenotype with *GALNTL5*.

**Table 2:** Validation of the IGs highlighted by our literature-based approach with the help of the 5 autism-related databases (AutismKB, SFARI, HuVarBase, DisGeNET and OpenTargets). Results highlighted in Bold were found in SFARI

INTERSECTION OF SUBPHENOTYPES AND 5 DATABASES					
SEVERITY ¯ SUBPHENOTYPE®	IQ	MEMORY	VERBAL	ATTENTION	LANGUAGE
INDEPENDENT SEVERITY	*NEMF*	*NEMF*	*NEMF*	*NEMF*	
SEVERE	***NIPA1***	***NIPA1***	***NIPA1***		***NIPA1***
MILD				***NIPA1***	
	*CELSR1*	*CELSR1*	*CELSR1*	*CELSR1*	
				*GALNTL5*	
				*MS4A14*	*MS4A14*
					***LRP2***
					***NAALADL2***
					***MUC12***
					***MCPH1***
					*EPN3*

### Functional analysis

As discussed in our methodology section, the genes highlighted by our two approaches were investigated regarding their functional role and their participation in biological processes. The results were grouped according to their function into 15 distinct categories: Developmental Biology, Nervous System Development, Synapses—Neurotransmission, Morphogenesis And Structure, Trafficking And Transport, Sensory, Cell Signaling, Cell Migration/Motility, Differentiation, Cell Cycle, Programmed Cell Death, Epigenetics, Metabolism, Post-Translational Modifications and Immunosystem.

**Fig. 6:**
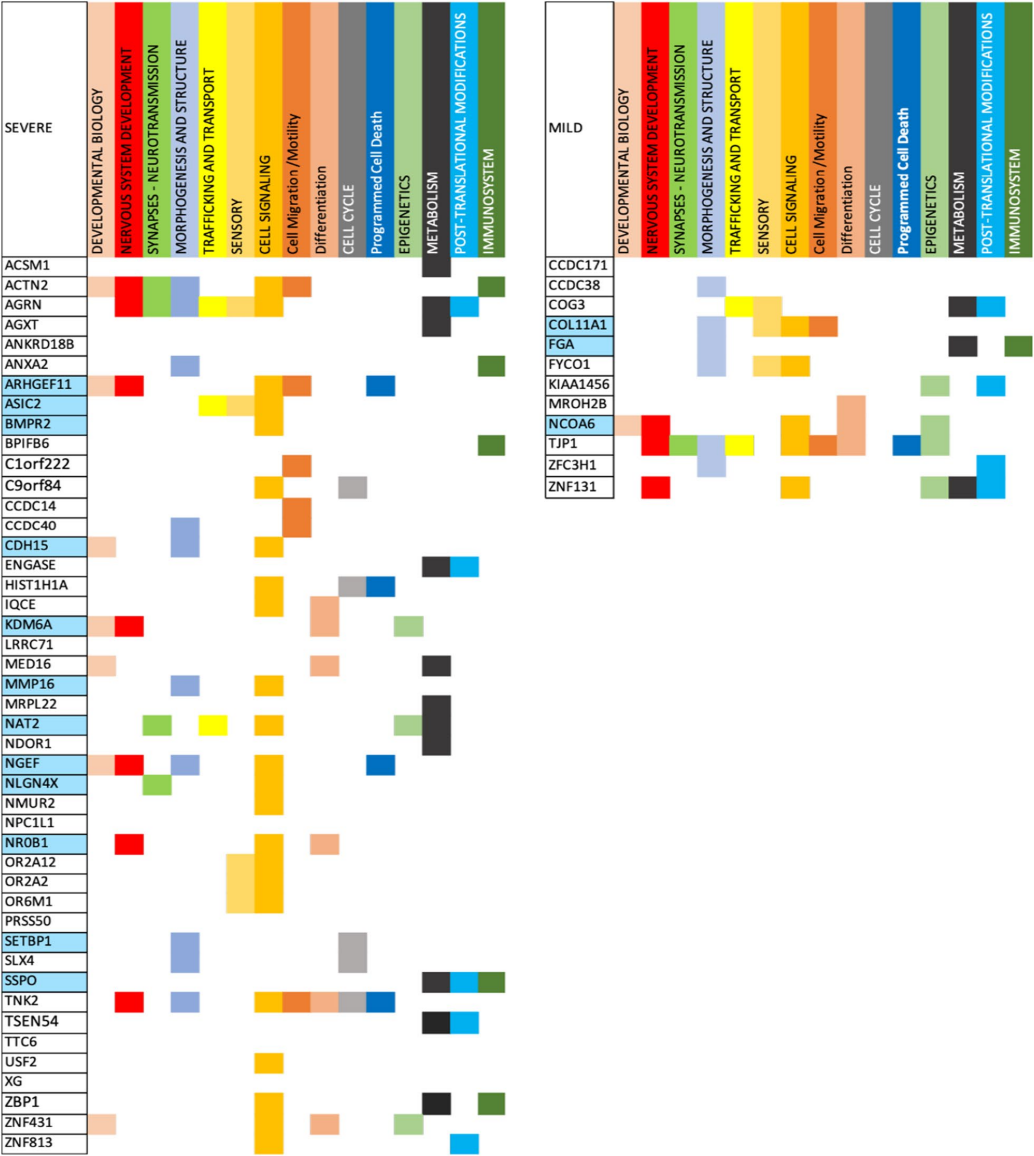
Functional analysis of the genes discovered by our Machine Learning approach for Severe and Mild autism. Figure shows individual gene participation in specific Autism Mechanisms (AMs)

For the genes highlighted by our machine learning approach, Fig. 6 showcases the Autism Mechanisms (AMs) implicated in severe and mild autism respectively. In total for the category Developmental Biology 7 genes in severe and only 1 in mild autism are involved. For the Nervous System Development 7 genes are involved in severe autism and 3 in mild. For Synapses—Neurotransmission 4 genes involved in severe and 1 in mild autism. For Morphogenesis and Structure 10 genes are involved in severe and 6 in mild. There are 3 genes for severe autism and 2 for mild involved in Trafficking And Transport, 5 genes for severe and 3 for mild in Sensory, 20 for severe and 5 for mild autism in Cell Signaling, Cell Migration/Motility, Differentiation, Cell Cycle, Programmed Cell Death, Epigenetics, Metabolism, Post-Translational Modifications and Immunosystem.

**Fig. 7:**
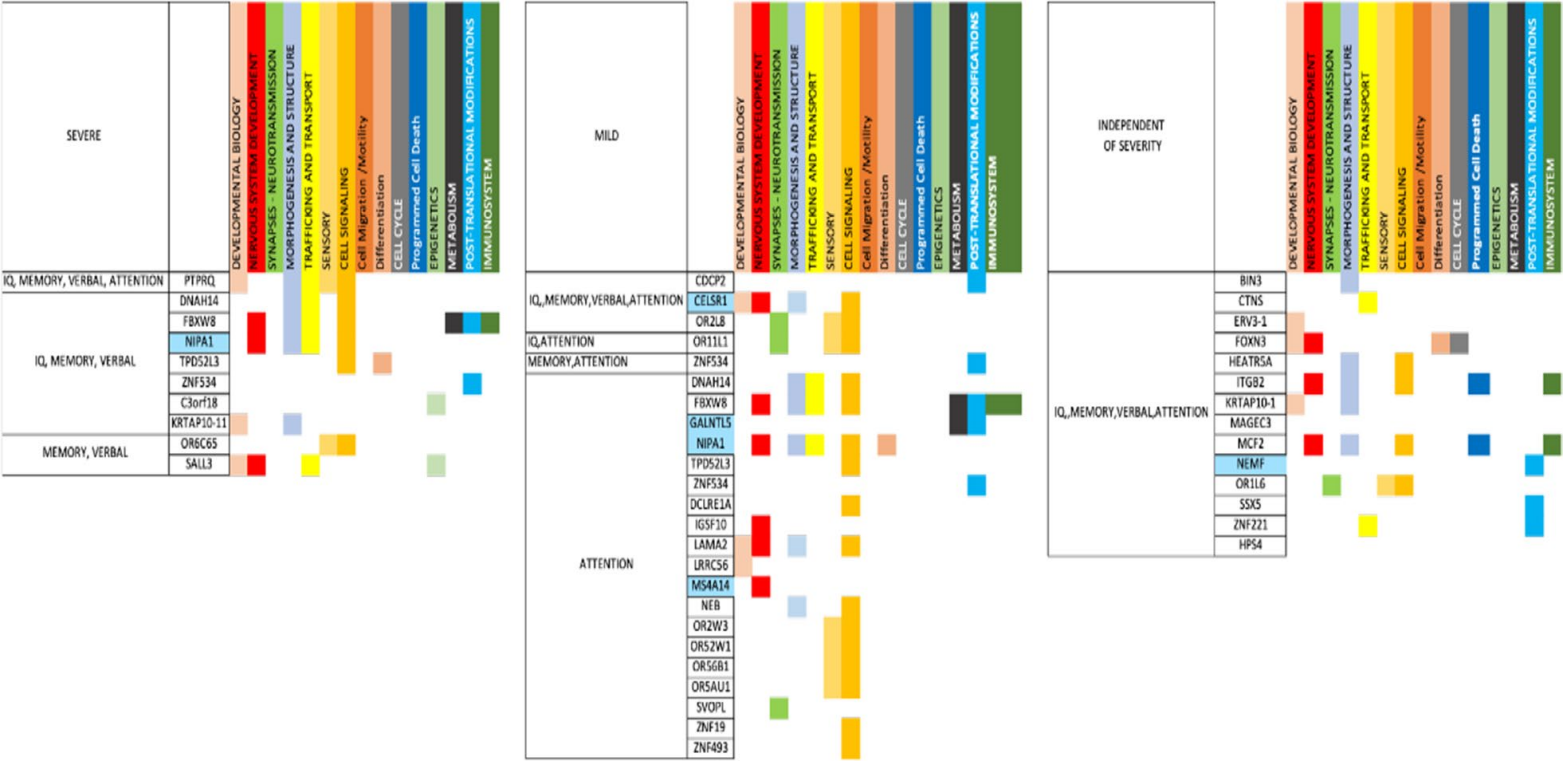
Functional analysis of the genes discovered by our literature-based approach for Severe and Mild autism broken down by specific clinical sub-phenotypes. Figure shows individual gene participation in specific Autism Mechanisms (AMs)

Breaking down the AMs brought to the foreground using our literature-based method (Fig. 7), there are 69 severity-independent AMs which span over all our categories except Epigenetics and Metabolism which appear to be severity-associated. In the severe autism group the AMs associated with IGs of “Language severe” and “IQ severe” are identical with the exception of the “Gene silencing” AM found only in the “IQ severe” due to the IG *C3orf18* and in total have 36 common AMs. Children in these groups also don’t appear to have AMs related to neurotransmission and cell cycle events. Likewise, the “Verbal severe” and “Memory severe” sample groupings are identical regarding their 51AMs (which is to be expected since they share the same IGs). There are no AMs associated with neurotransmission and cell cycle processes in these 2 groups, The “Attention severe” AMs are all related to the *PTPRQ* IG which is the only severity-dependent IG in the group. *PTPRQ* is linked with developmental, morphogenic, sensory and signaling processes.

In our mild autism sub-phenotypical groupings only the “Memory mild” and “Verbal mild” are completely alike. These include 23 AMs in total which are associated with general and nervous system development, neurotransmission and morphogenesis. The “IQ mild” IGs are involved in 25 AMs which do not include any associated with trafficking and transport, cell cycle, epigenetic, metabolic or immunological processes. The “Attention mild” IGs are involved in 41 AMs from all our categories but do not include any epigenetic modifications. Finally, the “Language mild” AMs are the most complex category spanning across 77 AMs from all our categories including the epigenetic histone phosphorylation.

In general, for both approaches many variants found in individual genes, like AGRN (which is involved in 9 functional modules in severe ASD), *ARHGEF11*, *NR0B1*, *NGEF*, *FOXN3*, *ITGF2* and *MCF2*, appear to be connected to a multitude of biological processes. Therefore, perturbations in any of these crucial genes, which have multiple functional involvements, may trigger the advent of disorders related to the structures and function of the CNS. It is also revealed that some genes like *HEATR5A*, *ITGF2*, *KRTAP10-1* and *MCF2* are involved in a single process like the morphogenesis and structure of synapses. Furthermore, we found mutational events in various proteins involved in sensory pathways which could explain the broad range of sensory abnormalities regularly observed in individuals across the autistic spectrum. Several of our findings highlighted perturbations in sensory and perceptual pathways which may explain impairments of attention, IQ, verbal ability and memory. For example, in all our severe autism samples (and none from the mild autism grouping) a common affected gene (*PTPRQ*) is found in all four clinical sub-phenotypes which is linked in literature to auditory impairment [55]. This gene can potentially serve as a biomarker of autism severity.

Deleterious/damaging variants in genes which encode signaling proteins can significantly alter the course of brain development, synaptic structure/ function and morphogenesis. For instance, the NLGN protein, found as significant in our results, plays an important role in synapsis and has been implicated by previous works in ASD [56]. In general, gene-encoding protein signaling is fundamental in neurodevelopment and post neurodevelopment processes such as synapse organization (*AGRN*, *TJP1*), cell migration (*ACTN2*, *TJP1*), axon guidance (*ACTN2*, *AGRN*, *TNK2*, *ARHGEF11*, *NGEF*, *FGA*) and dendrite development (*AGRN*), and any perturbation in processes like these may trigger the rise of disorders related to the structures and functions of the CNS. Also, *BMPs*, whose signaling has been shown to be dysregulated in ASD, constitute the largest subdivision of the *TGF-β* superfamily and are critical in the development of the CNS.

## Discussion

Autism is a neurodevelopmental disorder with heterogeneous genomic and phenotypical characteristics. There is also a high hereditary factor involved in its presentation but also a discrepancy regarding the sex of patients with a known 4 to 1 male to female ratio which is also present in the current study [57]. Our design assesses rare genetic risk variations in ASD to predict symptom severity based on genetic variation but also studies the perturbed gene effects on autism-related clinical observations linking the phenotypical to the genetic variation of ASD. This link of heterogeneity could involve many types of variables. Our observations distinguish clinical severity on a variety of characteristics like IQ, memory, attention, and verbal ability with the IQ range having the highest impact. These observations are strong candidate sources of etiologic differences and were carried out in a real-life setting, enabling us to determine baseline information for the bioinformatics approaches. The latter include both a machine learning and a literature-based technique, in order to validate and substantially extend current knowledge on ASD phenotypical severity but also individual characteristics.

Current criteria for clinical classification of ASD individuals provide a valuable behavioral depiction of the disorder but often fall short when grading them into severe vs. non-severe target groups. In this study, we use a WES dataset of 33 children with ASD. Given the small sample size of our dataset we focus more on the potential of these methodologies for disorder classification and the novelty of some of the identified genes. Using a machine-learning algorithm, based on linear regression polygenic risk score assessment, we select informative genes with the potential to contribute toward the grading of ASD. We obtain specific molecular signatures from severe and non-severe subtypes of autistic samples and show that these molecular signatures have the potential to define prognostic subclasses. Further experimentation is required before the role of the genes and variants can be deemed diagnostic. These subclasses involve in total 48 genes linked to severe ASD out of which only 12 have been previously identified and 12 genes linked to mild ASD with only 3 represented in current databases. We further show that 28/84 identified variants were found to underline specific functional classes related to autism and intellectual disability, as well as other disorders. Polygene risk score grading of samples using top variants is in agreement to prior clinical grading for our dataset with 81.81% prediction accuracy; thus, showing that our model is capable of recapitulating the clinical diagnostic methodology employed for this small number of children. However, we further show that six samples were particularly challenging to diagnose molecularly. This can be attributed to a variety of reasons, including methodological or biological factors which can always contribute to variation during experiments. Moreover, as seen in Fig. 5 we observe that there is a specific genetic profile that extends to all children with ASD in the severe subtype for IQ, memory and verbal skills (and in the vast majority of children for attention skills) as well as to all children in the mild subtype for attention. These results are in line with our phenotypical data for visual and auditory attention skills and in accordance with the literature, where both visual and auditory attention disorders have been found for mild and severe phenotypes in ASD [58–60]. These results also suggest that a high percentage of children present Attention Deficit Hyperactivity Disorder (ADHD) symptoms which might be due to either comorbidity or due to a common underlying factor [61].

In addition, this work employs a linear regression machine-learning model grading of these samples into molecular subtypes. Our approach allows for the extraction of genomic signatures from the bipartite (severe vs. non-severe) autistic classification scheme used to train our risk model. These signatures show differences in prognosis when compared to clinical grading showing valuable additive information that is impossible to obtain form clinical diagnosis alone. We envision that the identification of the novel set of variants and genes underlying these molecular signatures will enable autistic diagnosis to progress toward a more quantitative realm, where individuals with ASD are viewed within an autistic spectrum rather than the categorical grouping into distinct subtypes. We emphasize that the interpretation of classification results based on genomic data must be accompanied by clinical annotation on as many levels as possible. Only by the integration of such work with expert clinical and pathological annotation, can we maximize the value of genotypic data, increase our understanding of autistic pathology, and further develop current diagnostic and therapeutic approaches.

To further extend this last point, we employed a literature-based approach taking into consideration known effects of genetic variations on the translated proteins. This highlighted 14 (13 novel and 1 known) autism-related genes, independent of phenotypical severity, which contain various severe protein changes. We believe that these genes constitute a genetic background which has the potential to characterize children with ASD. Clusters of novel genes carrying “deleterious/damaging” variants are found to signify different degrees of severity. Furthermore, a plethora of genes have been linked to specific disorder manifestations (sub-phenotypes) namely IQ, memory, attention and verbal impairments and can help elucidate the symptomatology of ASD’s severity. These genes have also allowed for ascertaining specific groupings of sub-phenotypes based on their common genetic signatures, like the memory and verbal traits which seem to have identical IGs in their severe state.

Both previously described bioinformatics approaches have allowed further function dissection and highlighting of important biological processes which bridge the gap between genotype and phenotype in autism. By analyzing the molecular profile based on the clinical severity of ASD as a whole and its individual core features, we identified several potential molecular signatures of disorder and symptom severity. Despite the complex architecture of mutational events associated with autism, the various proteins involved, appear to converge on common processes such as synaptic function, brain development, chromatin remodelers (epigenetics processes), cell life processes, morphology/structures and function, sensory and signaling pathways. The autism-related core features which arise from underlying vulnerabilities are related to pleiotropic genes which associate with important molecular mechanisms.

Several observations regarding gene participation in specific and multiple biological processes were made, covering a wide range of functions.

Macroscopically, there is a high diversity of pathway groupings in our results. It is important to underline that there are considerably more genes perturbed in biological processes which relate to the CNS and neurodevelopment when examining the severe side of autism. There is also strong indication of higher variant occurrence in severe ASD where we observe that in CNS development there are 8 genes (*ACTN2*, *AGRN*, *TNK2*, *ARHGEF11*, *NGEF*, *KDM6A*, *AGRN*, *NR0B1*) highlighted in severe cases and only 4 (*TJP1*, *ZNF131*, *NCOA6*, *FGA*) in mild, in synapsis and neurotransmission 4 (*AGRN*, *ACTN2*, *NLGN4X*, *NAT2*) in severe versus only 1 (*TJP1*) in mild among other examples. Our findings also support the idea that ASD-associated genes may contribute not only to core characteristics of ASD but also potentially enhance vulnerability to other systemic problems including metabolic conditions, immune system dysfunction etc. something that is also previously described in literature [62].

We hereby acknowledge that our study has some limitations. The sample is small and thus our classification approach is mostly centered on the prediction and verification of these genes and does not hold a diagnostic nature per se. Moreover, the genes identified should be treated with caution again due to the limited sample size of our dataset.

## Conclusions

In conclusion, this study utilizes machine learning classification tools to obtain novel genes implicated in mild/moderate or severe ASD symptoms by constructing SNP-based classification models with 82% prediction. Our de novo implicated ASD risk genes appear to provide a substantial extension of previously reported genes, enriching current ASD-gene and variant databases. These risk genes can potentially be used to distinguish children with different degrees of ASD symptom severity, however substantial further experimentation is required to fully validate their diagnostic capacity. We also provide further clarification of the relationship between ASD risk mutations and intellectual disability [low on intelligence quotient—(IQ)], and impairment in memory, verbal disturbances and attention deficits. We believe that this study will help bridge the genotype-to-phenotype gap in ASD, illuminating how genetic variation can drive the severity of the disorder and/or specific pathological traits exhibited by individuals with ASD. By predicting the disorder’s severity genetically, children with ASD could receive more targeted care.

## Data Availability

All data are available on request from the authors.

## Appendix

See Tables 3 and 4.

**Table 3:** Groups’ performance on IQ, vocabulary, memory and attention tasks

Variables	ASD_MH		ASD_L		n	U	p	g
	Mean	SD	Mean	SD				
Non-verbal IQ: RPM Standard Score	98.61	15.13	40.66	12.23	33	0.00***	< 0.001	4.17
Expressive Vocabulary: CVS Standard Score	76.11	12.43	3.33	12.91	33	0.00***	< 0.001	5.75
Picture comprehension: DSLD Test Preschool	–	–	2.57	2.95	15	–	–	–
Attention (total score): TAAC Test Percentile	6.33	10.32	0.07	0.26	33	13.00***	< 0.001	.82
Auditory Attention: TAAC Test Percentile	16.66	26.21	0.14	0.35	33	24.00***	< 0.001	.85
Visual Attention: TAAC Test Percentile	10.94	18.47	0.13	0.35	33	7.00***	< 0.001	.79
Visual Attention Range: TAAC Test Percentile	17.67	23.84	0.33	1.29	33	20.50***	< 0.001	.98
VSTM Sentence recall: TAAC Test Percentile	14.28	14.63	0.67	0.26	33	11.50***	< 0.001	1.26
VSTM Word Recall: Memory Test	20.39	7.09	1.07	4.13	33	4.00***	< 0.001	3.25
Immediate Visual Memory: Memory Test	24.44	9.80	4.07	5.23	33	13.50***	< 0.001	2.53
Delayed Visual Memory: Memory Test	4.05	2.34	0.53	1.12	33	24.00***	< 0.001	1.86
Visual Information Recall: Memory Test	6.05	4.64	0.47	0.83	33	32.00***	< 0.001	1.60
Narration Total Elements: Memory Test	5.50	5.54	0.07	0.26	33	25.50***	< 0.001	1.32
Narration Total Sections: Memory Test	4.94	4.99	0.07	0.26	33	32.00***	< 0.001	1.32
Information Retention Factor: Memory Test	5.28	6.06	0.40	1.55	33	32.00***	< 0.001	1.06
Recognition: Memory Test	19.67	6.68	1.00	3.87	33	10.00***	< 0.001	3.34

*RPM* Raven Progressive Matrices, *CVS* Crichton Vocabulary Scale, *DSLD Test Preschool* Detection of Speech and Language Disorders Test Preschool, *TAAC* Test for the Assessment of Attention and Concentration in Primary School

^**^*p* < 0.01, ****p* < 0.001; Hedges' g: 0.2 = small effect size, 0.5 = medium effect size, 0.8 = large effect size

**Table 4:** Total of 84 unique top variants for severe vs non-severe molecular classification selected from LOOCV

rs_ids	gene	chrom	start	end	aa_change	impact	aaf_1kg_all
rs76264143	*AGRN*	chr1	982843	982844		intron variant	0.04
rs2748972	*C1orf222*	chr1	1891476	1891477	S/P	missense variant	0.13
rs1763347	*COL11A1*	chr1	103354427	103354428	G	synonymous variant	0.62
rs12119908	*LRRC71*	chr1	156902221	156902222	R/H	missense variant	0.20
rs822431	*LRRC71*	chr1	156902280	156902281	S/A	missense variant	0.28
rs4570419	*ARHGEF11*	chr1	156907030	156907031		intron variant	0.22
rs2275199	*ARHGEF11*	chr1	156909694	156909695	N	synonymous variant	0.19
rs2275206	*ARHGEF11*	chr1	156939066	156939067		splice region variant	0.18
rs12063382	*ACTN2*	chr1	236925843	236925844	S	synonymous variant	0.20
rs1061157	*BMPR2*	chr2	203421198	203421199	R	synonymous variant	0.12
rs2921705	*NGEF*	chr2	233792564	233792565		intron variant	0.17
rs34116584	*AGXT*	chr2	241808313	241808314	P/L	missense variant	0.08
rs66494441	*AGXT*	chr2	241808462	241808463		intron variant	-1.00
rs34726174	*AC104809.3*	chr2	241871846	241871847	G/R	missense variant	0.13
rs4683158	*FYCO1*	chr3	46010076	46010077	R/Q	missense variant	0.93
rs4682801	*FYCO1*	chr3	46021217	46021218	R	synonymous variant	0.76
rs12492868	*PRSS50*	chr3	46755936	46755937	T	synonymous variant	0.38
rs34788938	*PRSS50*	chr3	46759009	46759010	Q/P	missense variant	0.31
rs56260729	*TNK2*	chr3	195594949	195594950	P/L	missense variant	0.12
rs2070018	*FGA*	chr4	155508626	155508627		intron variant	0.89
rs2271704	*MROH2B*	chr5	41008779	41008780	L/P	missense variant	0.78
rs316408	*ZNF131*	chr5	43066773	43066774		upstream gene variant	0.67
rs3749787	*NMUR2*	chr5	151784182	151784183	L	synonymous variant	0.23
rs7965	*MRPL22*	chr5	154346324	154346325	K	synonymous variant	0.06
rs2251702	*C6orf229*	chr6	24797646	24797647	H	synonymous variant	0.52
rs62000984	*HIST1H1A*	chr6	26017674	26017675	L	synonymous variant	0.09
rs2969042	*IQCE*	chr7	2612293	2612294		intron variant	0.26
rs2969043	*IQCE*	chr7	2612294	2612295		intron variant	0.26
rs10264715	*NPC1L1*	chr7	44555405	44555406	Y	synonymous variant	0.08
rs34947817	*OR2A12*	chr7	143792990	143792991	S/N	missense variant	0.11
rs10230228	*OR2A2*	chr7	143806687	143806688	Q/K	missense variant	0.16
rs10252253	*OR2A2*	chr7	143807303	143807304	L/P	missense variant	0.16
rs7791767	*SSPO*	chr7	149513151	149513152		non coding transcript exon variant	0.21
rs10250401	*SSPO*	chr7	149515102	149515103		non coding transcript exon variant	0.20
rs622106	*KIAA1456*	chr8	12878676	12878677	A	synonymous variant	0.71
rs3739310	*KIAA1456*	chr8	12878806	12878807	C/G	missense variant	0.65
rs503550	*KIAA1456*	chr8	12879061	12879062	L	synonymous variant	0.71
rs608909	*KIAA1456*	chr8	12879333	12879334	V	synonymous variant	0.71
rs608052	*KIAA1456*	chr8	12879538	12879539	R/G	missense variant	0.70
rs7826836	*KIAA1456*	chr8	12888907	12888908	S/A	missense variant	0.67
rs1799931	*NAT2*	chr8	18258369	18258370	G/E	missense variant	0.08
rs16892543	*MMP16*	chr8	89340161	89340162	P	synonymous variant	0.21
rs1328285	*CCDC171*	chr9	15922136	15922137		intron variant	0.88
rs62559879	*ANKRD18B*	chr9	33566233	33566234	A	synonymous variant	0.16
rs11791445	*C9orf84*	chr9	114476747	114476748	M/L	missense variant	0.14
rs12352352	*C9orf84*	chr9	114484782	114484783	P	synonymous variant	0.14
rs10512411	*C9orf84*	chr9	114490228	114490229	A	synonymous variant	0.14
rs73563696	*NDOR1*	chr9	140108856	140108857	S	synonymous variant	0.14
rs76301014	*OR6M1*	chr11	123676387	123676388	R/C	missense variant	0.12
rs1298463	*ZFC3H1*	chr12	72013831	72013832	A	synonymous variant	0.51
rs6538681	*CCDC38*	chr12	96284649	96284650	A	synonymous variant	0.75
rs2985989	*COG3*	chr13	46108853	46108854	L	synonymous variant	0.81
rs12586727	*TTC6*	chr14	38218342	38218343	I/V	missense variant	0.21
rs2229518	*TJP1*	chr15	30008888	30008889	A	synonymous variant	0.79
rs12904906	*ANXA2*	chr15	60689994	60689995		intron variant	0.11
rs714181	*SLX4*	chr16	3640273	3640274	P/L	missense variant	0.24
rs3743690	*ACSM1*	chr16	20635417	20635418	K	splice region variant	0.18
rs2301672	*ACSM1*	chr16	20636813	20636814	S	synonymous variant	0.18
rs12922670	*CDH15*	chr16	89234947	89234948		upstream gene variant	0.06
rs9890913	*ASIC2*	chr17	31618550	31618551	L	synonymous variant	0.13
rs77247739	*TSEN54*	chr17	73518327	73518328	Q/P	missense variant	0.07
rs3744183	*ENGASE*	chr17	77075666	77075667	I	synonymous variant	0.42
rs3744184	*ENGASE*	chr17	77075669	77075670	P	synonymous variant	0.41
rs3744185	*ENGASE*	chr17	77075672	77075673	P	synonymous variant	0.42
rs3744186	*ENGASE*	chr17	77076439	77076440	S	synonymous variant	0.39
rs3752042	*CCDC40*	chr17	78010412	78010413		upstream gene variant	0.12
rs11082414	*SETBP1*	chr18	42529995	42529996	V/L	missense variant	0.16
rs78047294	*MED16*	chr19	871986	871987	T	synonymous variant	0.17
rs17445374	*ZNF431*	chr19	21326357	21326358	D/G	missense variant	0.14
rs2280746	*USF2*	chr19	35770055	35770056	V/I	missense variant	0.20
rs12104393	*CCDC114*	chr19	48801217	48801218		intron variant	0.15
rs2242463	*CCDC114*	chr19	48806976	48806977	D	synonymous variant	0.15
rs28582401	*CCDC114*	chr19	48807366	48807367	L	synonymous variant	0.15
rs2617667	*ZNF813*	chr19	53993669	53993670	A/T	missense variant	0.30
rs17373408	*BPIFB6*	chr20	31624299	31624300	S	synonymous variant	0.07
rs2070326	*BPIFB4*	chr20	31678533	31678534	L	synonymous variant	0.22
rs3787220	*NCOA6*	chr20	33337750	33337751	P	synonymous variant	0.80
rs6060043	*NCOA6*	chr20	33364583	33364584		intron variant	0.81
rs4811888	*ZBP1*	chr20	56182182	56182183	Q/E	missense variant	0.13
rs5939319	*XG*	chrX	2700156	2700157	D/N	missense variant	0.04
rs7049300	*NLGN4X*	chrX	5821785	5821786	T	synonymous variant	0.12
rs6150	*NR0B1*	chrX	30327366	30327367	C	synonymous variant	0.10
rs5952285	*KDM6A*	chrX	44913051	44913052		intron variant	0.24
rs5952682	*KDM6A*	chrX	44966794	44966795		intron variant	0.24

## Abbreviations

ASD: Autism spectrum disorder
IQ: Intelligence quotient
DSM-V: Diagnostic and statistical manual of mental disorders, 5th edition
NGS: Next-generation sequencing
WES: Whole exome sequencing
WGS: Whole genome sequencing
GWAS: Genome-wide association studies
CNVs: Copy number variants
SNVs: Single-nucleotide variants

## References

[1] Association AP (2013). Diagnostic and statistical manual of mental disorders (DSM-5®).

[2] Luyster RJ (2008). Language assessment and development in toddlers with autism spectrum disorders. J Autism Dev Disord.

[3] Thurm A (2007). Predictors of language acquisition in preschool children with autism spectrum disorders. J Autism Dev Disord.

[4] Tonnsen BL (2016). Prevalence of autism spectrum disorders among children with intellectual disability. Am J Intellect Dev Disabil.

[5] D’Abate L (2019). Predictive impact of rare genomic copy number variations in siblings of individuals with autism spectrum disorders. Nat Commun.

[6] Lord C, Veenstra-VanderWeele J (2016). Following the trail from genotype to phenotypes. JAMA Psychiat.

[7] Pinto D (2014). Convergence of genes and cellular pathways dysregulated in autism spectrum disorders. Am J Hum Genet.

[8] Sanders SJ (2015). Insights into autism spectrum disorder genomic architecture and biology from 71 risk loci. Neuron.

[9] Hanson E (2015). The cognitive and behavioral phenotype of the 16p11.2 deletion in a clinically ascertained population. Biol Psychiatry.

[10] Isles AR (2016). Parental origin of interstitial duplications at 15q11.2–q13.3 in schizophrenia and neurodevelopmental disorders. PLoS Genet.

[11] Frohlich J (2016). A quantitative electrophysiological biomarker of duplication 15q11.2–q13.1 syndrome. PLoS ONE.

[12] Moreno-De-Luca A (2015). The role of parental cognitive, behavioral, and motor profiles in clinical variability in individuals with chromosome 16p11.2 deletions. JAMA Psychiat.

[13] Chung WK (2021). 16p11.2 deletion syndrome. Curr Opin Genet Dev.

[14] Conran CA (2016). Population-standardized genetic risk score: the SNP-based method of choice for inherited risk assessment of prostate cancer. Asian J Androl.

[15] Kypreou KP (2016). Prediction of melanoma risk in a Southern European population based on a weighted genetic risk score. J Investig Dermatol.

[16] Paila U (2013). GEMINI: integrative exploration of genetic variation and genome annotations. PLoS Comput Biol.

[17] E.P., Consortium (2012). An integrated encyclopedia of DNA elements in the human genome. Nature.

[18] Kent WJ (2002). The human genome browser at UCSC. Genome Res.

[19] Amberger JS (2015). OMIM.org: Online Mendelian Inheritance in Man (OMIM®), an online catalog of human genes and genetic disorders. Nucleic Acids Res.

[20] Sherry ST (2001). dbSNP: the NCBI database of genetic variation. Nucleic Acids Res.

[21] Kanehisa M, Goto S (2000). KEGG: kyoto encyclopedia of genes and genomes. Nucleic Acids Res.

[22] Prasad TS, Kandasamy K, Pandey A (2009). Human Protein Reference Database and Human Proteinpedia as discovery tools for systems biology. Methods Mol Biol.

[23] Karczewski KJ (2017). The ExAC browser: displaying reference data information from over 60 000 exomes. Nucleic Acids Res.

[24] Auton A (2015). A global reference for human genetic variation. Nature.

[25] Landrum MJ (2018). ClinVar: improving access to variant interpretations and supporting evidence. Nucleic Acids Res.

[26] Forbes SA (2017). COSMIC: somatic cancer genetics at high-resolution. Nucleic Acids Res.

[27] Kircher M (2014). A general framework for estimating the relative pathogenicity of human genetic variants. Nature.

[28] Adzhubei IA (2010). A method and server for predicting damaging missense mutations. Nat Methods.

[29] Kumar P, Henikoff S, Ng PC (2009). Predicting the effects of coding non-synonymous variants on protein function using the SIFT algorithm. Nat Protoc.

[30] Raven J, Rust J, Squire A (2008). Manual for coloured progressive matrices and crichton vocabulary scale.

[31] Sideridis GD, Antoniou F, Mouzaki A, Simos P (2015). The Greek version of Raven’s Colored progressive matrices and crichton vocabulary scale.

[32] Simos P, Mouzaki A, Sideridis G (2007). Test of the assessment of attention and concentration in primary school.

[33] Besevegis E, Economou A, Milonas Κ (2007). Memory test.

[34] Oikonomou A, Bezevegis I, Milonas K, Varlokosta S (2007). Screening tool for the detection of speech and language disorders for preschoolers.

[35] Li H, Durbin R (2009). Fast and accurate short read alignment with Burrows–Wheeler transform. Bioinformatics.

[36] Li H (2009). The sequence alignment/map format and SAMtools. Bioinformatics.

[37] McKenna A (2010). The Genome Analysis Toolkit: a MapReduce framework for analyzing next-generation DNA sequencing data. Genome Res.

[38] Purcell S (2007). PLINK: a tool set for whole-genome association and population-based linkage analyses. Am J Hum Genet.

[39] Kundu S (2011). PredictABEL: an R package for the assessment of risk prediction models. Eur J Epidemiol.

[40] Pencina MJ, D'Agostino RB, Demler OV (2012). Novel metrics for evaluating improvement in discrimination: net reclassification and integrated discrimination improvement for normal variables and nested models. Stat Med.

[41] Gelman A, et al., Package ‘arm’. Data Analysis Using Regression and Multilevel/Hierarchical Models, 2015.

[42] Wang M, Zhao Y, Zhang B (2015). Efficient test and visualization of multi-set intersections. Sci Rep.

[43] Venny OJ. An interactive tool for comparing lists with Venn Diagrams. http://bioinfogp.cnb.csic.es/tools/Venny/index. 2007.

[44] Xu L-M (2012). AutismKB: an evidence-based knowledgebase of autism genetics. Nucleic Acids Res.

[45] Abrahams BS (2013). SFARI Gene 2.0: a community-driven knowledgebase for the autism spectrum disorders (ASDs). Mol. Autism.

[46] Ganesan K (2019). HuVarBase: A human variant database with comprehensive information at gene and protein levels. PLoS ONE.

[47] Piñero J (2017). DisGeNET: a comprehensive platform integrating information on human disease-associated genes and variants. Nucleic Acids Res..

[48] Carvalho-Silva D (2019). Open Targets Platform: new developments and updates two years on. Nucleic Acids Res.

[49] Joshi-Tope G (2005). Reactome: a knowledgebase of biological pathways. Nucleic Acids Res.

[50] Safran M, Dalah I, Alexander J, Rosen N, Iny Stein T, Shmoish M, et al. GeneCards Version 3: the human gene integrator. Database (Oxford). 2010;2010:baq020. 10.1093/database/baq020. 10.1093/database/baq020 PMC2938269 20689021

[51] Szklarczyk D (2014). STRING v10: protein–protein interaction networks, integrated over the tree of life. Nucleic Acids Res.

[52] U., Consortium (2015). UniProt: a hub for protein information. Nucleic Acids Res.

[53] Smith CL, Eppig JT (2009). The mammalian phenotype ontology: enabling robust annotation and comparative analysis. Wiley Interdiscip Rev Syst Biol Med.

[54] Ashburner M (2000). Gene Ontology: tool for the unification of biology. Nat Genet.

[55] Schraders M (2010). Mutations in PTPRQ are a cause of autosomal-recessive nonsyndromic hearing impairment DFNB84 and associated with vestibular dysfunction. Am J Hum Genet.

[56] He L (2018). Role of NRXN-NLGN-SHANK pathway gene variations in the pathogenesis of autism spectrum disorders. Zhonghua Yi Xue Yi Chuan Xue Za Zhi.

[57] Rapin I (2009). Subtypes of language disorders in school-age children with autism. Dev Neuropsychol.

[58] Chien Y (2015). Visual memory and sustained attention impairment in youths with autism spectrum disorders. Psychol Med.

[59] Corbett BA, Constantine LJ (2006). Autism and attention deficit hyperactivity disorder: assessing attention and response control with the integrated visual and auditory continuous performance test. Child Neuropsychol.

[60] Sturm H, Fernell E, Gillberg C (2004). Autism spectrum disorders in children with normal intellectual levels: associated impairments and subgroups. Dev Med Child Neurol.

[61] Christakou A (2013). Disorder-specific functional abnormalities during sustained attention in youth with attention deficit hyperactivity disorder (ADHD) and with autism. Mol Psychiatry.

[62] Voineagu I (2011). Transcriptomic analysis of autistic brain reveals convergent molecular pathology. Nature.

